# Potent antimicrobial activity of hydrogel loaded with the antimicrobial peptide, D-Bac8c^2,5 Leu^, against monospecies and polymicrobial biofilms of *Staphylococcus aureus* and *Pseudomonas aeruginosa*

**DOI:** 10.3389/fmicb.2025.1571649

**Published:** 2025-04-24

**Authors:** Hawraa Shahrour, Daniela Alves Ferreira, Luke Sheridan, Deirdre Fitzgerald-Hughes, James P. O’Gara, Marc Devocelle, Helena Kelly, Eoghan O’Neill

**Affiliations:** ^1^Department of Clinical Microbiology, RCSI Education and Research Centre, Beaumont Hospital, RCSI University of Medicine and Health Sciences, Dublin, Ireland; ^2^School of Pharmacy and Biomolecular Sciences, RCSI University of Medicine and Health Sciences, Dublin, Ireland; ^3^Department of Microbiology, School of Biological and Chemical Sciences, University of Galway, Galway, Ireland; ^4^Department of Chemistry, RCSI University of Medicine and Health Sciences, Dublin, Ireland; ^5^Department of Microbiology, Connolly Hospital, Dublin, Ireland

**Keywords:** antimicrobial resistance, biofilm, chronic wounds, antimicrobial peptides (AMP), dressings

## Abstract

**Introduction:**

Acute and chronic wound infections involving biofilms and caused by antimicrobial resistant (AMR) pathogens present significant challenges in healthcare, leading to substantial patient morbidity, increased hospital stays, and rising healthcare costs. Novel antimicrobial therapies are urgently needed to address these infections.

**Methods:**

A screening of multiple antimicrobial peptides (AMPs) was performed and the most potent candidate, D-Bac8c^2,5 Leu^, was tested against monospecies and polymicrobial biofilms of *Staphylococcus aureus* and *Pseudomonas aeruginosa* using static and dynamic *in vitro* models. Cytotoxicity was evaluated on human cell lines, and the peptide was incorporated into a methylcellulose hydrogel to assess sustained release and antimicrobial efficacy as a hydrogel dressing.

**Results:**

D-Bac8c^2,5 Leu^ significantly reduced biofilm viability in both monospecies and polymicrobial biofilms. In static biofilm assays, treatment led to a 2–3 log reduction in bacterial load compared to untreated controls. In Duckworth biofilm flow device, a similar reduction was observed, demonstrating efficacy in conditions mimicking wound environments. Furthermore, D-Bac8c^2,5 Leu^ exhibited low cytotoxicity against human cell lines, and its incorporation into a methylcellulose hydrogel facilitated sustained release and enhanced antimicrobial activity. Furthermore, the peptide-loaded hydrogel showed considerable efficacy in disrupting pre-formed biofilms, underscoring its potential as a novel treatment for acute and chronic wound infections.

**Discussion:**

These findings highlight the potential of D-Bac8c^2,5 Leu^ to help address the urgent need for effective therapies against AMR pathogens and biofilm-associated wound infections. Further studies should focus on *in vivo* efficacy to optimize its therapeutic application in wound care.

## 1 Introduction

Both acute and chronic wound infections are a cause of considerable patient morbidity and are pervasive across the healthcare settings, spanning community to hospital settings. They encompass a spectrum of conditions, including surgical site infections (SSIs), burns wound infections, and skin infections post-trauma (Monahan et al., 2020; Ding et al., 2022). Wound infections pose a substantial burden on healthcare systems worldwide, leading to prolonged hospitalizations, increased healthcare expenditures, and diminished quality of life for affected individuals. Moreover, the prevalence of chronic wounds is expected to surge worldwide driven by the increase of lifestyle diseases, such as diabetes, obesity, and cardiovascular diseases (Sen, 2021; Hurlow and Bowler, 2022). Complicating matters is the emergence of antimicrobial resistance (AMR), exemplified by priority pathogens like methicillin-resistant *Staphylococcus aureus* and multi-drug-resistant Gram-negative organisms as identified by the World Health Organization (Kuthyar et al., 2021).

The problem of AMR is compounded by the widespread involvement of bacterial biofilms in healthcare-associated infections including wound infections (Bjarnsholt, 2013; Evelhoch, 2020; Assefa and Amare, 2022). Data from the National Institutes of Health and the Center for Disease and Prevention, indicate that a substantial majority (65–80%) of human infections are caused by biofilms, where it is also reported that most chronic wounds contain biofilms (Donlan, 2002; Wu et al., 2019). Notably, biofilms harbor a high frequency of antibiotic tolerant persister cells, up to 1,000-fold more resistant to antibiotics than their planktonic counterparts, which play an important role in chronic and relapsing infections and have been shown to facilitate the emergence of AMR (Tran et al., 2023; Wang and Jin, 2023). Traditional management, such as surgical debridement, skin grafting and systemic antibiotics, often fall short in achieving satisfactory healing outcomes for chronic wounds in particular (Evelhoch, 2020; Thaarup et al., 2022). These factors highlight the urgent need for effective wound management strategies that address both the clinical and economic implications of wounds while navigating the threat posed by AMR (Falcone et al., 2021).

In this landscape, antimicrobial peptides (AMPs) have emerged as promising treatment alternatives, endowed with potent antimicrobial properties which are often effective against antibiotic-resistant strains, coupled with immunomodulatory effects and low toxicity profiles (Shahrour et al., 2019; Pontes et al., 2022). Beyond their broad-spectrum antimicrobial efficacy, AMPs exhibit multifaceted biological activities including the regulation of inflammatory response and the promotion of re-epithelialisation, which accelerate the wound healing process (Luo and Song, 2021; Miao et al., 2021). Researchers have recently introduced AMPs into dressings used for wound healing and have shown enhanced antimicrobial and healing properties (Khosravimelal et al., 2021; Wei et al., 2021; Yang et al., 2021). Among these dressings are hydrogels, which offer advantage such as their user-friendly nature and ability to promote wound healing while minimizing scarring (Thapa et al., 2020; Zhang et al., 2022; Jeong et al., 2023; Zhou et al., 2023).

In this study we screened the antibiofilm potential of a number of AMPs with recognized antimicrobial properties which were identified following literature review. Using *in vitro*, *ex vivo*, and *in vivo* models of wound infection, the ability of the most promising AMP, D-Bac8c^2,5 Leu^, to disrupt mono species and polymicrobial biofilms associated with acute and chronic wound infections was measured before and after its incorporation into a hydrogel system.

## 2 Materials and methods

### 2.1 Peptide synthesis

The peptides sequences, D-Bac8c^2,5 Leu^, DRGN-1, 1,037, IDR-1018, and RP557, were synthesized as previously described (Forde et al., 2014, 2016). Peptides were synthesized by solid-phase 9-fluorenylmethoxycarbonyl (Fmoc) chemistry, using a CEM Liberty Blue Automated Microwave Peptide Synthesizer (CEM Corporation, Buckingham, UK) and purified to a purity of 95–99% using reverse-phase high-performance liquid chromatography (HPLC). Peptides’ were characterized by matrix-assisted laser desorption ionization-time-of-flight (MALDI-TOF) mass spectrometry (MS). The peptides’ sequences and characteristics are represented in Table 1.

**Table 1:** Molecular characteristics of the tested peptides.

AMP	Amino acid sequence (Peptide amides)^a^	Molecular weight (g/mol)	Net charge	References
D-Bac8c^2,5 Leu^	RLWVLWRR	1183.47	+4	Forde et al. (2014, 2016)
DRGN-1	PSKKTKPVKPKKVA	1535.95	+7	Chung et al. (2017)
1,037	KRFRIRVRV	1228.54	+7	de la Fuente-Núñez et al. (2012)
IDR-1018	VRLIVAVRIWRR	1535.93	+5	Reffuveille et al. (2014)
RP557	RFCWKVCYKGICFKKCK	2139.73	+7	Woodburn et al. (2019)

^aUppercase characters represent L-amino acids; lowercase characters represent D-amino acids.^

### 2.2 Bacterial strains and growth conditions

Strains used in the study were isolates of methicillin-resistant *Staphylococcus aureus* (MRSA); USA300 LAC, BH1CC (O’Neill et al., 2007), methicillin-sensitive *S. aureus* (MSSA); SH1000 (Horsburgh et al., 2002) and BH48 (O’Neill et al., 2007); and *Pseudomonas aeruginosa* PAO1 (ATCC 156920). Clinical isolates were obtained from a previous collection of isolates of infected wounds. Bacteria were grown aerobically at 37°C with shaking at 200 rpm in Brain Heart Infusion (BHI, Oxoid, Ireland). Selective media, Mannitol Salt Agar (SLS, Ireland) and MacConkey agar (SLS, Ireland), for re-isolation of bacterial strains from mixed species biofilms were used. Bolton Broth (Fannin, Ireland) supplemented with 50% bovine heparin plasma (TebuBio, UK), and 5% laked horse blood (Sigma-Aldrich, Ireland) was used to mimic *in vivo* conditions for static antibiofilm testing. For dynamic biofilm growth Simulated Wound Fluid (SWF) prepared as described previously with slight modification, 3% v/v fetal bovine serum in maximum recovery diluent (Merck, Ireland) was used (Barbour et al., 2016).

### 2.3 Antimicrobial susceptibility testing

Minimum inhibitory concentration (MIC) was determined aerobically using the broth microdilution assay as recommended by the Clinical and Laboratory Standards Institute (CLSI) (CLSI, 2018). Briefly, 2-fold serial dilutions of the antimicrobial peptides of interest and conventional antibiotics were prepared in Mueller-Hinton broth (MHB) (Merck, Ireland) in a 96-well round bottom polypropylene plate (ThermoFisher, Ireland). Diluted bacterial suspensions in sterile PBS (Sigma, Ireland) were added to each well resulting in a final cell density of approximately 10^5^ colony forming units (CFU)/mL. Microplates were incubated statically for 18 h at 37°C. In each assay, inoculated medium without antimicrobials and uninoculated medium served as growth and sterility controls, respectively. Conventional antibiotics, gentamicin (Merck, Ireland) and mupirocin (SLS, UK), were tested as comparator antimicrobials. The MIC value was defined as the lowest antimicrobial concentration showing no visible growth relative to positive (no antimicrobial agent) and negative controls (BHI alone). Experiments were conducted in triplicate, and independent replicates were performed three times to yield *n* = 3.

### 2.4 Static biofilm formation

Biofilms were formed under static conditions using flat 96-well polystyrene plates as described previously with few modifications (Nasreddine et al., 2018). Briefly, overnight cultures in BHI were washed and diluted 1:200 in fresh Bolton Broth media supplemented with 50% bovine heparin plasma, and 5% laked horse blood. From this suspension, 100 μL was used to inoculate the microtiter plate wells prior to static incubation at 37°C for 24 h, 3 days and 5 days. The media in each well was changed daily during prolonged incubation periods (3 and 5 days). Following the initial incubation period and washing, 100 μL of D-Bac8c^2,5 Leu^, was added to each test well at 37°C for 6 h at a concentration range of 64–256 μg/mL. After incubation with D-Bac8c^2,5 Leu^, biofilms were washed twice with PBS and biofilm assessment was performed. For the polymicrobial biofilms, a bacterial solution ratio 1:1 of *S. aureus* and *P. aeruginosa* at 1 × 10^5^ CFU/mL each was used to inoculate the wells. In each assay, inoculated medium without antimicrobials and uninoculated medium served as growth and sterility controls, respectively. Experiments were conducted in triplicate, and independent replicates were performed three times to yield *n* = 3.

### 2.5 Resazurin conversion assay

Viability of biofilm cells after treatment was measured using resazurin-conversion assay as previously described (Hogan et al., 2016) (Sigma-Aldrich, Ireland). Briefly, 100 μL of resazurin solution at 88 μM in sterile water was added to biofilms in each well andplates were incubated for 1 hat 37°C, protected from light. Fluorescence intensity was detected at excitation of 544 nm and emission of 590 nm using a fluorimeter, Perkin Elmer 2030 Multilabeled Reader, Victor 3X. Adenosine triphosphate (ATP)-dependent conversion of the nonfluorescent resazurin into the fluorescent resorufin as a measure of bacterial viability is proportional to the number of living cells present. Experiments were conducted in triplicate, and independent replicates were performed three times to yield *n* = 3.

### 2.6 Assessment of biofilm viable cells by enumeration of colony forming units

After treatment, formed biofilms were washed twice with sterile PBS and then suspended in TrypLE solution (Gibco, Dublin, Ireland). A serial of 10-fold dilutions of the suspended biofilms were performed in 96-well flat bottom microplates. Then 10 μL of each dilution was plated in triplicate on MH agar using the drop method. For polymicrobial biofilms, dilutions were plated on Mannitol Salt Agar and MacConkey Agar. After overnight incubation at 37°C, CFU were counted and results were expressed as log_10_CFU/mL.

### 2.7 Biofilm formation under dynamic conditions

#### 2.7.1 Enumeration of colony forming units method

The Duckworth biofilm flow device was set-up and run following the method described by Duckworth et al. (2018). Twelve disks of agar at 1.5% (w/v) concentration were cut using a 10 mm French press punch and placed into designated wells in the flow device. Cellulose filter (13 mm, 0.22 μm, Merck Millipore, Ireland) was placed on top of each agar disk, and was inoculated with 10 μL of the bacterial inoculum. The device was placed in an incubator at 37°C while a continuous flow of SWF was pumped into the device at a flow rate of 0.322 mL/min for 24 h or 5 days using a peristaltic pump (Supplementary Figure S2). Subsequently, topical treatment with D-Bac8c^2,5 Leu^ was introduced and allowed to run for a further 6 h. The biofilm formed on the cellulose filters were collected and suspended in 1 mL PBS. Colony counting was performed as described above.

#### 2.7.2 Confocal microscopy method

The Duckworth biofilm flow device was prepared and run as described above with slight modification. A solution of agarose dissolved in SWF (0.3 g in 20 mL), mixed with bacterial cultures at 10^8^ CFU/mL and then supplemented with 3% FBS and 0.15% collagen (Merck, Ireland) was allowed to solidify at room temperature and then 12 disks of that was cut using a 10 mm French press punch. The disks were then placed into designated wells and experiments run as previously described. After treatment, thin slices of the agarose disks were cut with a sterile scalpel and stained using bacterial viability LIVE/DEAD Backlight kit (ThermoFisher Scientific, Ireland) according to the manufacturer’s protocol. Imaging of the disks was performed using a confocal laser scanning microscope (Cell Observer Z1 micro- scope, Zeiss, Oberkochen, Germany) equipped with a 63 Xobjective. Image acquisition was done with the Zeiss software package, and image processing with ImageJ (ImageJ/Fiji 1.46, National Institution of Health, USA). Three representative images were obtained per sample group per experiment.

### 2.8 Investigation of the potential induction of antimicrobial resistance *in vitro*

This assay was performed as outlined previously with some modifications (Ge et al., 1999; Mohan et al., 2019). Briefly, the *in vitro* serial passage study involved exposing bacteria diluted to 10^4^ CFU/mL in cationic adjusted Muller Hinton Broth (Merck, Ireland) to D-Bac8c^2,5 Leu^ at one-half of the previously established bactericidal concentration. After 90 min of incubation at 37°C, 10 μL was plated in triplicate on MH agar and incubated again overnight. Post-incubation, surviving colonies were harvested, diluted in PBS and re-adjusted to 1 × 10^4^ CFU/mL before repeated treatment with the sub-bactericidal concentration. This concentration was determined prior to the initiation of the study and after the 4th, 7th, 11th, and 14th passages. If the concentration after these passages was greater than the original determinate, then at passages 5, 6 and 7, the amount of peptide used during the bacterial challenge was increased. This was continued over a 14-day course. This assay was performed in independent duplicates.

### 2.9 Effect of D-Bac8c^2,5 Leu^ on mammalian cells viability

Cellular viability was performed in human THP-1 cell line by a colorimetric assay using tetrazolium dye, 3-(4-5-dimethylthiazol-2-yl)-2,5-diphenyltetrazolium bromide (MTT; Sigma-Aldrich, Ireland). THP-1 were grown in RPMI medium supplemented with 10% heat-inactivated fetal bovine (FBS). At 90% confluence, cells were centrifuged and diluted to a 1:5 ratio with complete RPMI. THP-1 cells were seeded in a 96 well plate at a density of 1 × 10^5^ cells/mL with increasing concentrations of D-Bac8c^2,5 Leu^. Cells were incubated for 24 h at 37°C with 5% CO_2_, 95% humidity. Post treatment, 10 μL of MTT was added (final concentration 500 μg/mL) to each well and incubated for 4 h at 37°C, 5% CO_2_. Formazan crystals were dissolved by the addition of 100% dimethyl sulfoxide (DMSO) and gentle shaking for 5 min at room temperature, in the dark. The absorbance was measured at 595 nm in a microplate spectrophotometer. Cell viability was calculated as a percentage of a control treated with PBS. Two technical replicates were performed each day on three independent days.

### 2.10 Haemolysis assays

Potential haemolytic activity of the peptide was determined using fresh human erythrocytes from healthy donors based on the method described by Cantisani et al. (2013). Human blood was drawn [ethylenediaminetetraacetic acid (EDTA); 1.6 mg/mL], and red blood cells (RBCs) were separated by centrifugation at 1000 x *g* for 5 min at 18°C. Upon 3 washes with PBS, the RBCs were further diluted 2-fold (v/v) into PBS. D-Bac8c^2,5 Leu^ was added to erythrocyte suspension at a final concentration ranging from 3.125 to 200 μg/mL in a final volume of 100 μL. Samples were incubated at 37°C for 24 h. The level of haemolysis was assessed by measuring the absorbance of supernatant at 490 nm. Triton-X-100 was used as a positive control for haemolysis.

### 2.11 Hydrogel formation

A previously established 2.5% (w/v) methylcellulose (MC) hydrogel was formulated with 5.6% (w/v) 𝛽-Glycerophosphate (GP) to induce thermoresponsivity. To ensure sterility, MC and deionized water were autoclaved using a standard autoclave cycle (121°C for 15 min), and the final formulation produced under aseptic conditions in a biosafety (BSC II) cabinet (ESCO LifeSciencesGroup, Singapore). D-Bac8c^2,5 Leu^ was added to final concentrations of 256 μg/mL and 512 μg/mL, and hydrogel without D-Bac8c^2,5 Leu^ was used as a negative control, for all tests.

### 2.12 Antimicrobial testing of free and loaded hydrogel

#### 2.12.1 Kill curve analysis

To conduct a kill curve analysis, bacterial cultures were prepared by resuspending pellets from overnight BHI cultures in MH broth and adjusting the bacterial density to 1 × 10^8^ CFU/mL. Peptide-loaded or peptide-free hydrogels were added to the bacterial cell suspension and incubated at 37°C with shaking for 6 ha. Samples were taken at hourly intervals, diluted, and the number of CFUs enumerated on MH agar to measure bacterial survival.

#### 2.12.2 Antibiofilm activity

Biofilm formation and treatment were conducted in 96-well plates as described in section 4.4. Briefly, test strains were inoculated into 96 well plates and incubated at 37°C for 1 day to allow biofilm formation. After incubation, planktonic cells were removed by washing with PBS, and the biofilms were then treated with either D-Bac8c^2,5 Leu^ hydrogel or a negative control for 6 h at 37°C. Post-treatment, the well contents were collected, resuspended in PBS, serially diluted, and plated on MH agar for viable cell analysis.

### 2.13 Statistics

Log_10_ transformation of CFU data was used to ensure a normal distribution. The data are presented as the mean ± SD. Statistical analyses were performed using GraphPad Prism version 9. Tests used for the *p*-value determination are mentioned in each figure legend.

## 3 Results

### 3.1 D-Bac8c^2,5 Leu^ has potent antimicrobial and antibiofilm activity against MRSA, MSSA, and *P. aeruginosa* biofilms

A comprehensive screen of five antimicrobial peptides (AMPs) (Table 1) was conducted to evaluate their effectiveness against a panel of 2 MRSA, 2 MSSA and 1 *P. aeruginosa* strains. Utilizing a high-throughput microtiter plate assay, we determined the MIC of each AMP and conventional antibiotics (Table 2). The results revealed distinct antimicrobial profiles among the tested peptides, with D-Bac8c^2,5 Leu^ and RP557 emerging as the most potent, exhibiting MIC values of 8 μg/mL and 16 μg/mL against *S. aureus* strains, respectively. Interestingly, variations in MICs were observed against PAO1, with D-Bac8c^2,5 Leu^ and RP557 displaying growth inhibition at 16 μg/mL and 8 μg/mL, respectively. Conversely, the five strains exhibited substantially lower levels of susceptibility to DRGN-1, 1,037, and IDR-1018 peptides (Table 2). Notably, D-Bac8c^2,5 Leu^, which exhibited the highest overall level of antimicrobial activity, also has the most attractive immunomodulatory profile (Zapotoczna et al., 2017). D-Bac8c^2,5 Leu^ was further tested against a collection of clinical isolates of *P. aeruginosa* and MRSA and MSSA *S. aureus.* The peptides showed effectiveness against all clinical wound isolates (Supplementary Table S1).

**Table 2:** Minimum inhibitory concentrations (MIC) of the tested antimicrobial agents against reference and clinical strains of *S. aureus* and *P. aeruginosa*.

Antimicrobial agent	MIC (μg/mL)				
	SH1000	BH48	USA300	BH1CC	PAO1
D-Bac8c^2,5 Leu^	8	8	8	8	16
DRGN-1	>400	>400	>400	>400	>400
1,037	128	128	128	128	64
IDR-1018	64	128	64	128	64
RP557	16	16	16	16	8
Fusidic acid	0.06	4	0.03	2	>512
Gentamicin	0.25	0.25	0.5	32	1
Mupirocin	0.03	0.03	0.06	2	>2048

**Figure 1:**
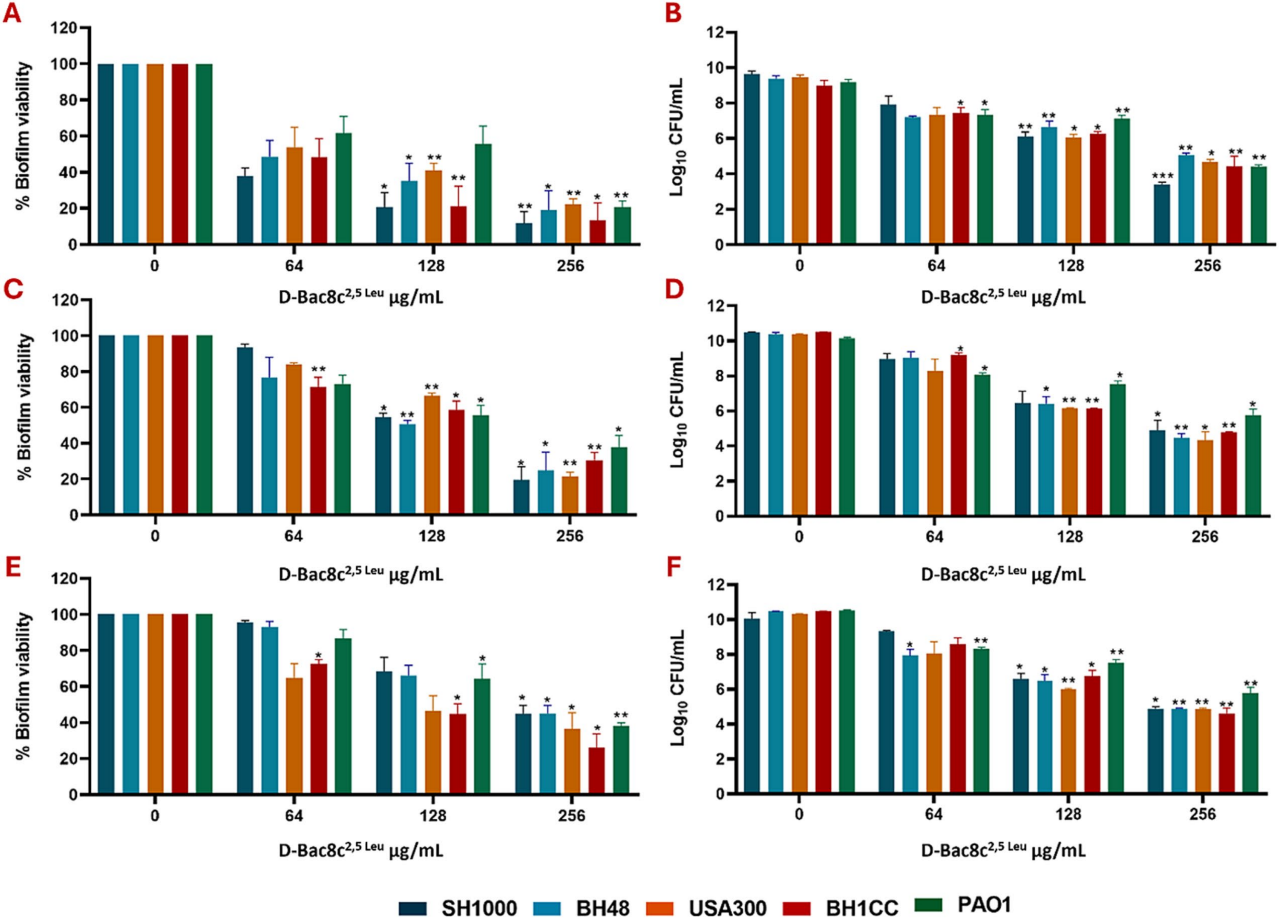
Efficacy of D-Bac8c^2,5 Leu^ free in solution against early and mature mono-species biofilms. Biofilms were formed for 1 day **(A,B)**, 3 days **(C,D)** and 5 days **(E,F)** under static conditions before treating them with increasing concentrations of D-Bac8c for 6 hat 37°C. Biofilm viability based on metabolic activity **(A,C,E)** and CFU/ml **(B,D,F)** was determined. Data are presented as mean ± SD of three independent experiments. Obtained results were analysed using two-way ANOVA and statistical differences were significant compared to the untreated control where *, ** and *** indicate a *p <* 0.05, 0.01 and 0.001, respectively.

**Figure 2:**
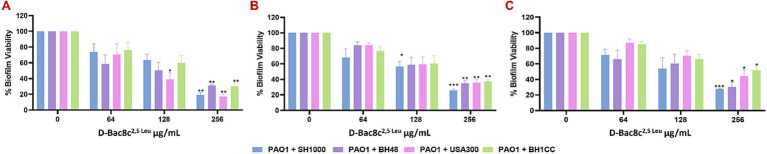
Efficacy of D-Bac8c^2,5 Leu^ free in solution against early and mature polymicrobial biofilms. Biofilms were formed for 1 day **(A)**, 3 days **(B)** and 5 days **(C)** under *in vivo* like *in vitro* conditions before treating them with increasing concentrations of D-Bac8c for 6 h at 37°C. Metabolic biofilm viability was detected and presented as mean ± SD of three independent experiments. Obtained results were analysed using two-way ANOVA and statistical differences were significant compared to the untreated control where *, ** and *** indicate a *p <* 0.05, 0.01 and 0.001, respectively.

Next, the antibiofilm activity of D-Bac8c^2,5 Leu^ was measured against preformed monospecies and polymicrobial biofilms of all five strains under both static and dynamic conditions, and within an *in vivo* like wound environment. Monospecies (Figure 1) and polymicrobial biofilms (Figure 2) were grown statically for 1, 3 and 5 days before a 6 h treatment with concentrations of D-Bac8c^2,5 Leu^ ranging from 64 (eight times the MIC) to 256 μg/mL (32 times the MIC). Results showed that the treated biofilms were significantly inactivated in a concentration-dependent manner. Enumeration of CFUs from polymicrobial biofilms treated with D-Bac8c^2,5 Leu^ further supported significant reductions in biofilm viability (Table 3).

**Table 3:** Efficacy of D-Bac8c^2,5 Leu^ against early and mature polymicrobial biofilms.

	Log change vs. untreated control					
	*S. aureus + P. aeruginosa*		*S. aureus + P. aeruginosa*		*S. aureus + P. aeruginosa*	
D-Bac8c^2.5 Leu^	64 μg/mL		128 μg/mL		256 μg/mL	
24 h biofilms						
PAO1 + SH1O00	−0.6	−0.64	−1.65*	−1.61*	−3. 37***	−3.78***
PAO1 + BH48	−0.84*	−0.29	−1.22**	−1.30***	−3. 37***	−2.64***
PAO1 + USA3O0	−0.72*	−0.65	−0.95**	−1.73***	−2.44***	−3.23***
PAO1 + BH1CC	−0.69	−0.6	−1.90***	−1.25*	−3.30***	−2.10***
3 days biofilms						
PAO1 + SH1000	−0.71	−0.88	−1.75**	−1.77**	−2.31***	−2.38***
PAO1 + BH48	−0.88	−1.29**	−1.85**	−1.99***	−2.26***	−2.68***
PAO1 + USA3O0	−0.4	−1.25*	−0.9	−1.62**	−1.89***	−2.48***
PAO1 + BH1CC	−0.66	−0.79	−1.31*	−1.24*	−1.77***	−2.19***
5 days biofilms						
PAO1 + SH1O00	−1.11*	−0.63	−2.13***	−2.06***	−2.90***	−2.76***
PAO1 + BH48	−0.93	−0.91	−1.59*	−2.25**	−2.00**	−2.85***
PAO1 + USA3O0	−0.9	−0.85	−1.68	−1.09	−2.88**	−2.94**
PAO1 + BH1CC	−0.98	−0.98	−1.50*	−1.54*	−2.65***	−2.58***

*^a^* Obtained results were analysed using two-way ANOVA and statistical differences were significant compared to the untreated control where *, ** and *** indicate a *p <* 0.05, 0.01 and 0.001, respectively.

**Figure 3:**
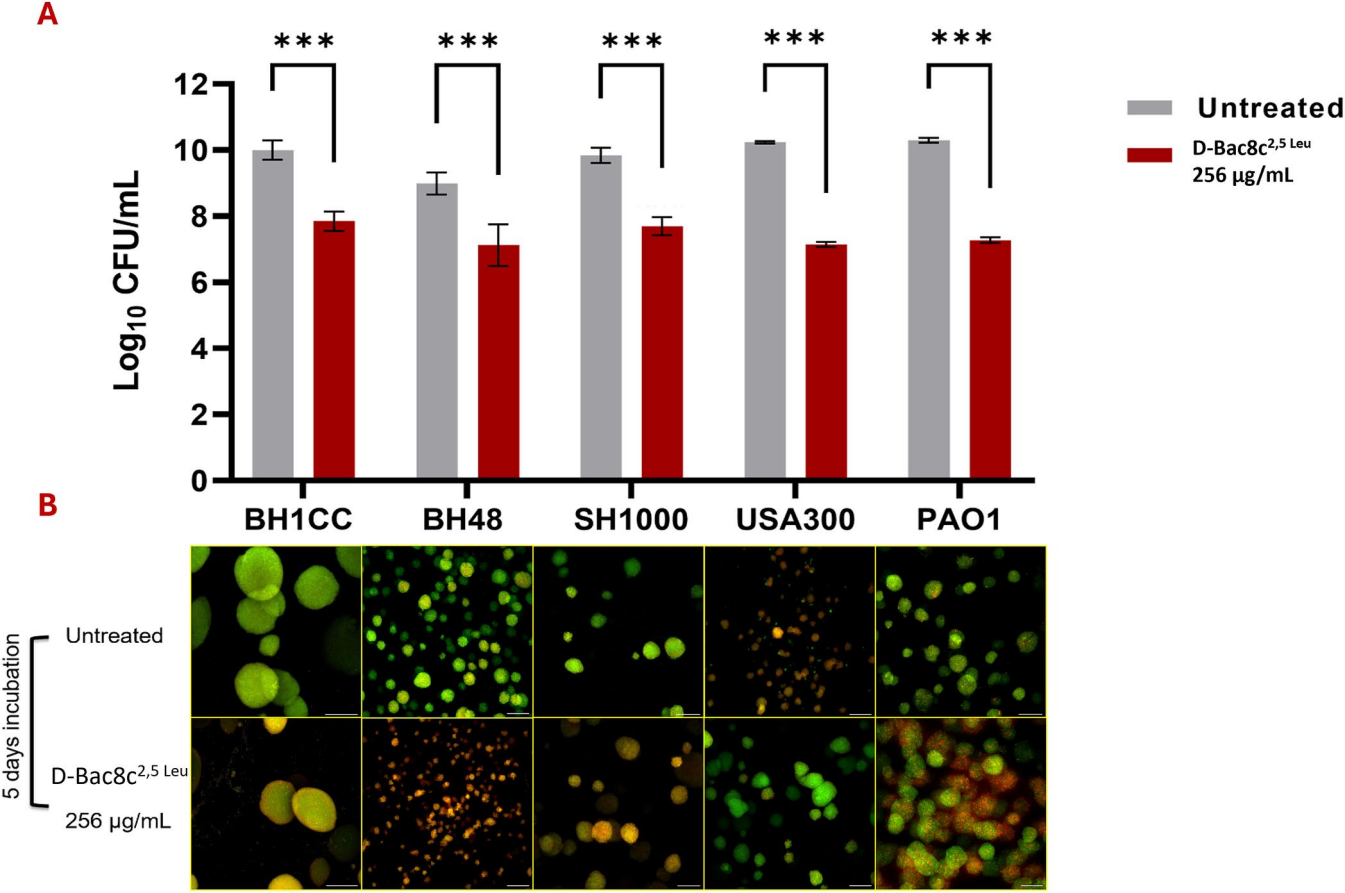
Antibiofilm activity of D-Bac8c^2,5 Leu^ against mono-species biofilms of *S. aureus* and *P. aeruginosa*. Biofilms were allowed to grow for 5 days in Duckworth system before treating them with the peptide. **(A)** Shows the number of viable cells present in the formed biofilm presented as Log_10_ CFU/mL ± SD. Analysis was done using a student *t*-test where the results were shown to be significantly different when comparing treated to untreated biofilm, *** indicating a *p* < 0.001. **(B)** Represents confocal microscopy data of treated and untreated biofilms (Green-stained cells correspond to viable cells and red-stained cells correspond to dead bacterial cells). Observations were shown to be reproduced in three independent experiments. Scale bar corresponds to 100 μm.

**Figure 4:**
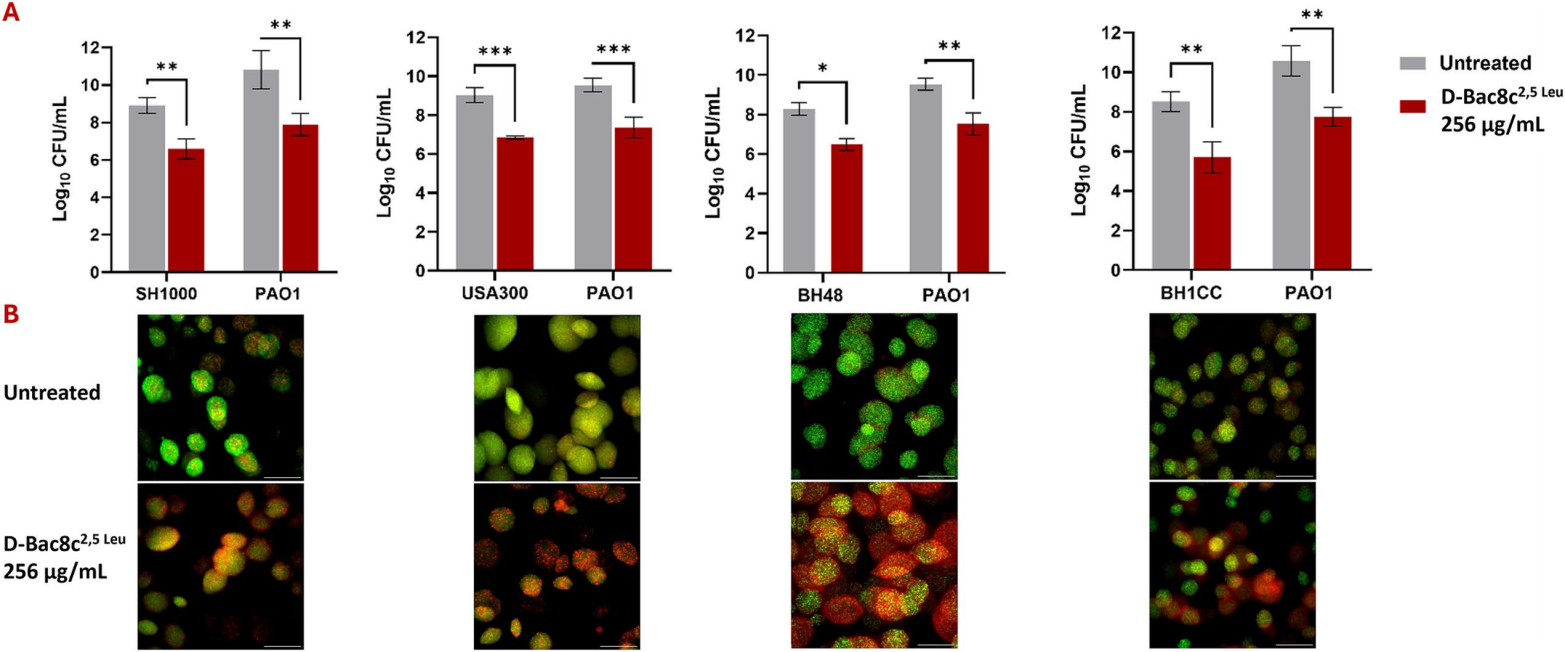
Antibiofilm activity of D-Bac8c^2,5 Leu^ against polymicrobial biofilms of *S. aureus* and *P. aeruginosa*. Biofilms were allowed to grow for 1 day in Duckworth system before treating them with the peptide. For each combination of *S. aureus* strain with PAO1, the graph **(A)** shows the number of viable cells present in the formed biofilm presented as Log_10_ CFU/mL ± SD and lower panel shows the corresponding confocal image. Analysis was done using a student *t*-test where the results were shown to be significantly different when comparing treated to untreated biofilm, *** indicating a *p* < 0.001. **(B)** Represents confocal microscopy data of treated and untreated biofilms (Green-stained cells correspond to viable cells and red-stained cells correspond to dead bacterial cells). Observations were shown to be reproduced in three independent experiments. Scale bar corresponds to 100 μm. * and ** indicate a *p <* 0.05, and 0.01, respectively.

Monospecies and polymicrobial biofilms grown for 1 and 5 days under shear flow conditions in the Duckworth device, subjected to treatment with D-Bac8c^2,5 Leu^ at 256 μg/mL for 6 h exhibited a significant reduction (up to a 3-log) in viability (Figures 3, 4). Furthermore, confocal microscopy, employing the LIVE/DEAD Backlight kit revealed a pronounced prevalence of dead cells (red) relative to viable cells (green) following treatment with the peptide, in both monospecies (Figure 3) and polymicrobial biofilms (Figure 4).

### 3.2 Hydrogel delivery of D-Bac8c^2,5 Leu^ enables topical application of the AMR

D-Bac8c^2,5 Leu^ at 256 μg/mL was incorporated into a 2.5% MC-5.6% *β*-GP hydrogel formulation. The 𝛽-GP-methylcellulose hydrogel exhibited strong thermoresponsive properties, transitioning from solid to a gel at approximately 34°C, as indicated by a sharp increase in storage modulus at this temperature (Supplementary Figure S1). When held at body temperature (37°C), the gel rapidly solidifies and maintains a high storage modulus over a 30-min period, demonstrating stability in physiological conditions. Additionally, the gel shows a reversible response to temperature fluctuations, with its storage modulus rising and falling as the temperature cycles between 37°C and 20°C, highlighting its adaptability to changing thermal environments. The incorporation of D-Bac8c^2,5 Leu^ into the gel did not alter its thermoresponsive profile (Supplementary Figure S1), indicating compatibility with bioactive agents.

**Figure 5:**
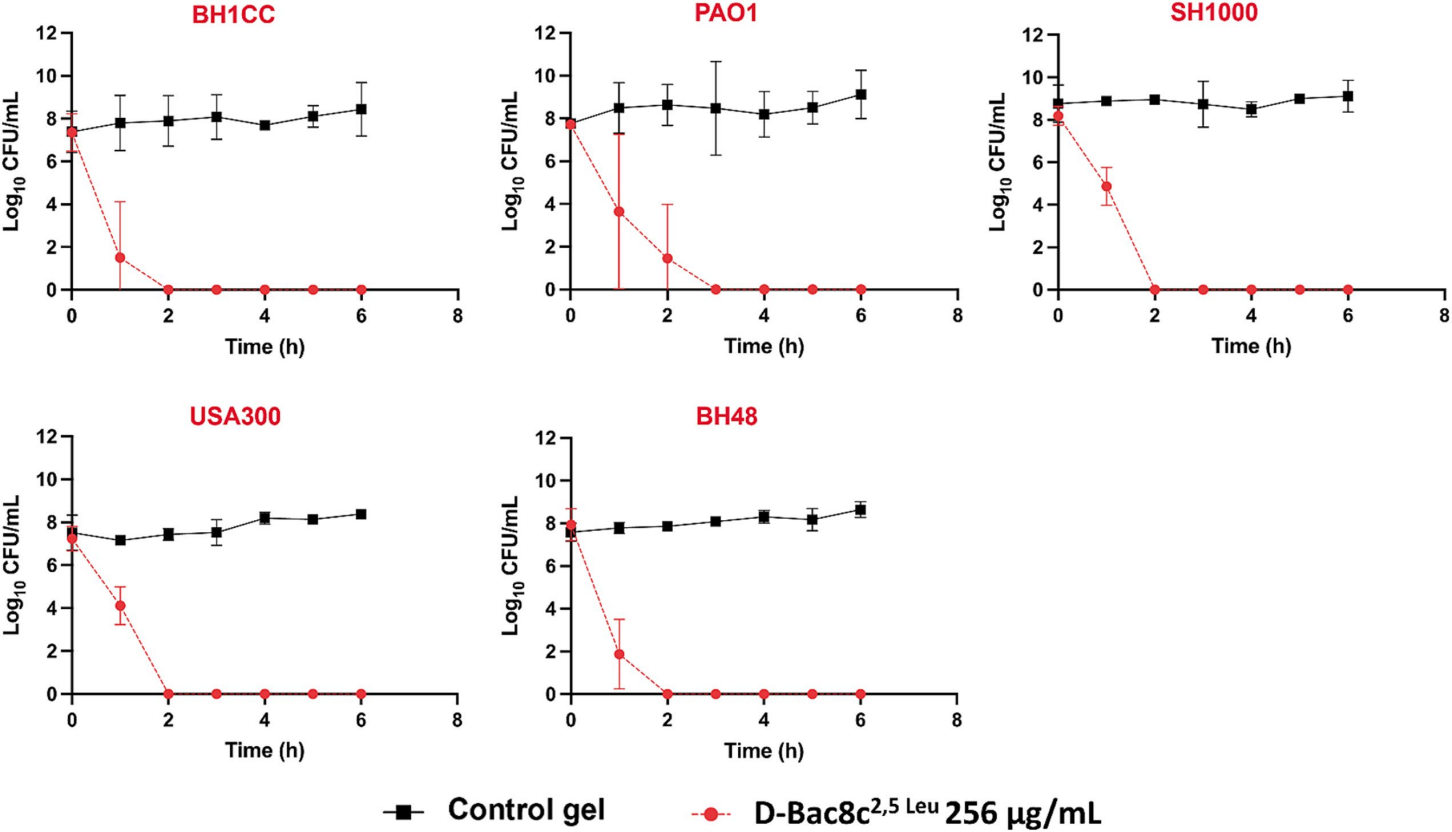
Kinetics of killing of planktonic bacterial cultures exposed to D-Bac8c^2,5 Leu^ at 256 μg/mL loaded hydrogel. Samples of cultures growing in presence of loaded and unloaded gels were taken at the indicated times post-inoculation and plated for viable counting. Results are the mean ± SD of 3 independent experiments performed in triplicate. Obtained results were analysed using Student *t*-test and statistical differences were significant (*p* < 0.05) compared to the control gel.

**Figure 6:**
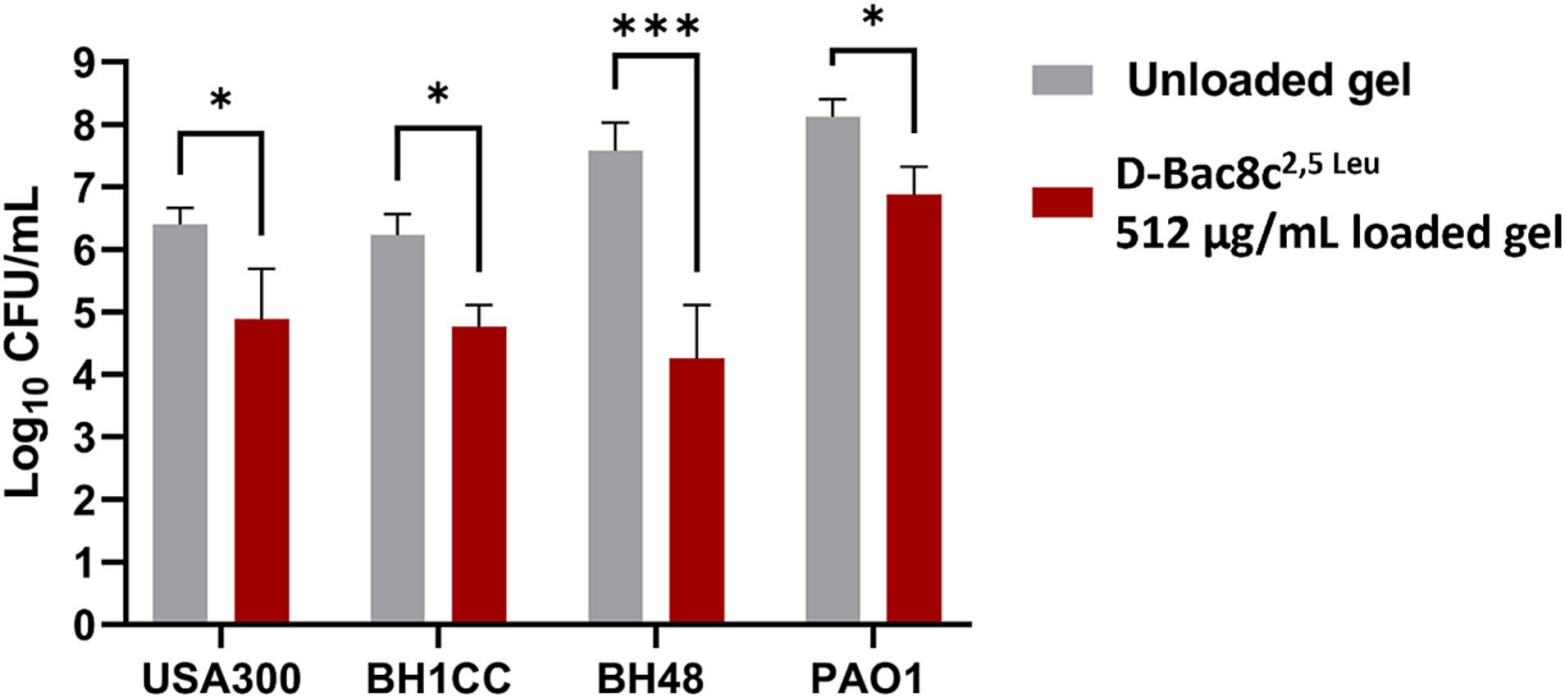
Biofilm Susceptibility of D-Bac8c^2,5 Leu^ at 512 μg/mL loaded hydrogel loaded hydrogel. Biofilms were formed on microtiter plates under wound-like environment for 24 h then treated with the gels for another 6 h. The number of viable cells in the wells was then determined and presented as Log_10_ CFU/mL ± SD. Analysis was done using a student *t*-test where the results were shown to be significantly different when comparing treated to untreated biofilms, * and *** indicate a *p <* 0.05, and 0.001, respectively.

A significant reduction in biofilm viability was measured following treatment with D-Bac8c^2,5 Leu^-loaded MC- β-GP hydrogel (at a concentration of 256 μg/mL) after just 1 h, with complete eradication after 2–3 h (Figure 5). Hydrogel loaded with D-Bac8c^2,5 Leu^ at 512 μg/mL achieved a 1.5–3 log reduction in biofilm viability after 6 h of treatment (Figure 6). A higher concentration of D-Bac8c^2,5 Leu^ was used in hydrogel used to eradicate biofilm to ensure that a minimum concentration of 64 μg/mL (Figures 1, 2 and Table 3) needed to inactivate biofilms, was released.

**Figure 7:**
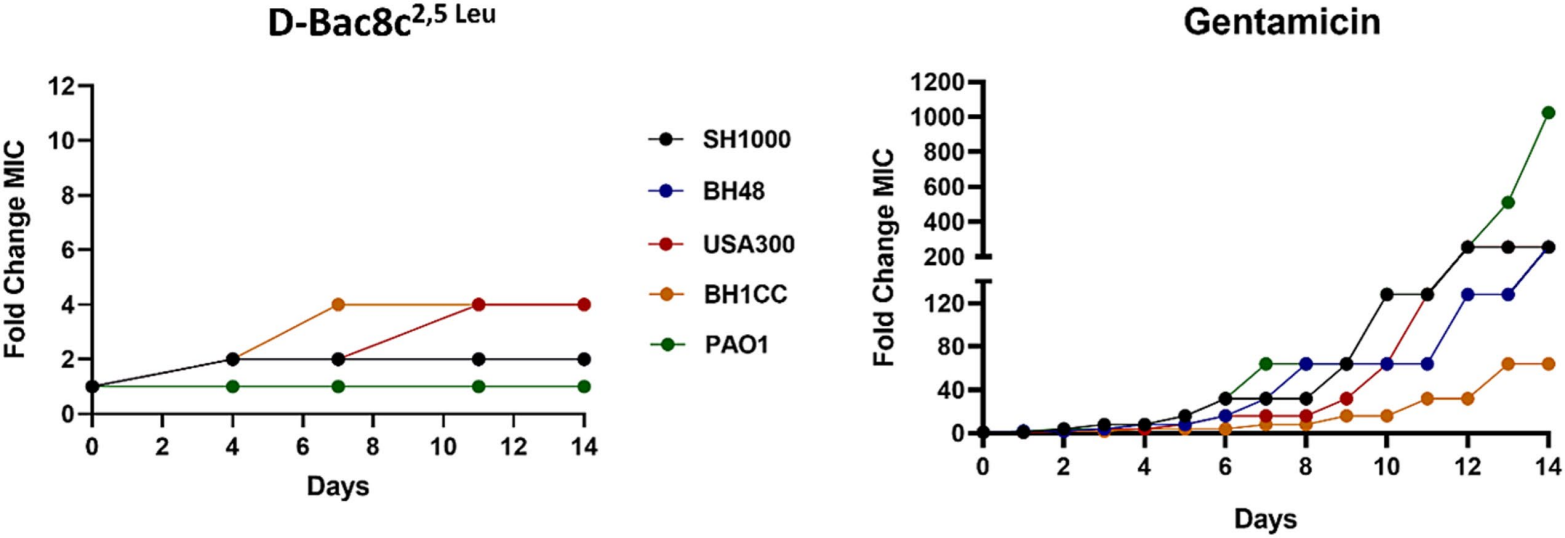
Assessment of the development of resistance to D-Bac8c^2,5 Leu^ peptide and Gentamicin antibiotic over 14 sequential passages. The data represents the fold change in MIC of Bac8c^2,5 Leu^ and Gentamicin after each passage over 14 days duration.

Finally, subculturing of the tested strains for 14 days in media supplemented with D-Bac8c^2,5 Leu^ at sub-MIC concentrations was not accompanied by increased resistance. As observed in Figure 7, compared to gentamicin, subculturing did not result in marked changes in MIC for three of the five strains. Relatively small fold changes compared to those found for gentamicin challenge were noted for BH1CC and USA300 strains after 8 days of exposure.

The cytotoxicity of D-Bac8c^2,5 Leu^ was investigated using human monocyte, keratinocyte and endothelial cell lines; THP-1, HaCaT and HUVEC cells, respectively. The 50% inhibitory concentration (IC50) was determined using an MTT assay and the results are presented in Table 4. The IC50 of the peptide against the human cell lines ranged between 134 and 427 μg/mL (Table 4), which is within the effective concentration of D-Bac8c^2,5 Leu^ against biofilms. Furthermore, exposure of human red blood cells to D-Bac8c^2,5 Leu^ for 1 h at 37°C resulted in negligible haemolytic activities compared to ethanol (Table 4).

**Table 4:** Effect of AMP D-Bac8c^2,5 Leu^ on the cellular viability of human cell lines.

IC50	HaCaT	HUVEC	THP-1	Haemolysis (hRBC^a^)
D-Bac8c^2,5 Leu^ μg/mL	426.7	134	331.7	414
Ethanol %	1.2	0.4	1	NA

Cells were exposed to the AMP for 24 h at 37°C, 5% CO_2_. Cell viability was assessed by MTT assay and IC50, the concentration of drug which exhibited 50% cell viability, was calculated. Data represents the average of three independent experiments. ^a^hRBC stands for human red blood cells.

## 4 Discussion

With the rise of AMR and the challenges posed by infections such as chronic wound infections, AMPs have emerged as promising candidates for use as novel antimicrobial agents (Xuan et al., 2023). Their broad-spectrum activity, rapid mode of action, and relatively low propensity for inducing resistance make them particularly appealing as therapeutic agents in the face of escalating AMR and difficult to treat infections (Magana et al., 2020; Yan et al., 2021). Their ability to disrupt and eradicate biofilms, a key virulence factor contributing to chronicity and treatment failure, underscores their potential as effective treatments for wound infections (Shahrour et al., 2019; Haidari et al., 2023). We screened a number of AMPs with recognised antibiofilm and antimicrobial properties against clinically relevant pathogens commonly associated with wound infections (de la Fuente-Núñez et al., 2012; Forde et al., 2014; Reffuveille et al., 2014; Chung et al., 2017a; Kizhakkedathu et al., 2019); D-Bac8c^2,5 Leu^ represented the most promising AMP to further investigate due to low broad-spectrum MICs against *S. aureus* and *P. aeruginosa* (Table 2 and Supplementary Table S1) and favorable cytotoxicity profile.

In this study we therefore, further investigated the antibiofilm and antimicrobial activity of D-Bac8c^2,5 Leu^ in a number of *in vivo* like models of wound infection. Under both static and dynamic conditions, D-Bac8c^2,5 Leu^ at a concentration of 256 μg/mL demonstrated a high potential for targeting mature biofilms formed for 1, 3 and 5 days. Mature biofilms more closely reflect the real-life clinical scenario in which a majority of patients invariably present to healthcare providers with chronic or non-healing wounds. Utilizing standardized 96-well plate biofilm assays, we observed a concentration-dependent reduction in biofilm metabolic activity. Experiments with the Duckworth biofilm flow device, which represents an *in vitro*-like *in vivo* wound infection model, provides a more clinically relevant assessment of the peptide’s efficacy against biofilms. By allowing biofilms to develop over 1 and 5 days with continuous flow of fresh SWF, this model better replicates the dynamic conditions present in acute and chronic wound infections. The observed significant reduction in viable counts of both monospecies and polymicrobial biofilms following treatment with D-Bac8c^2,5 Leu^ underscores its effectiveness in eradicating bacterial biofilms commonly encountered in chronic wounds. Furthermore, the confirmation of these findings through confocal microscopy, which revealed a dominance of dead cells in D-Bac8c^2,5 Leu^-treated biofilms compared to mostly viable cells in untreated controls, provides compelling evidence of the peptide’s ability to disrupt biofilm integrity and induce bacterial cell death. Due to its potent antibiofilm activity, D-Bac8c^2,5 Leu^ remains a promising alternative for treating persistent infections, even at higher concentrations. Unlike conventional antibiotics, which may fail even at doses 50 times the MIC, its ability to disrupt biofilms enhances its therapeutic potential against resistant microbial communities (Tran et al., 2023).

Our study also addressed concerns regarding the development of bacterial resistance to D-Bac8c^2,5 Leu^, with the peptide maintaining its susceptibility profile even after prolonged exposure. This is particularly significant given the escalating global concern over AMR and the urgent need for alternative therapies. The methodology employed, involving continuous passaging of bacterial strains in the presence of sub-MIC concentrations of D-Bac8c^2,5 Leu^, reflects a realistic scenario of prolonged exposure to the peptide in a clinical setting. Despite the sub-lethal concentration used, which was insufficient to induce bacterial killing, the study aimed to assess the potential for bacteria to develop resistance over time. The observation that all tested strains remained susceptible to D-Bac8c^2,5 Leu^, even after continuous exposure for 14 days, suggests a low likelihood of resistance emergence (Figure 7).

According to our previous study D-Bac8c^2,5 Leu^ demonstrates promising biocompatibility and suitability for human use in other clinical applications, such as device-associated infections (Zapotoczna et al., 2017). The MTT assays reported in that paper indicate that D-Bac8c^2,5 Leu^ exhibits low cytotoxic effects on human umbilical vein endothelial cells (HUVECs) and human keratinocytes (HaCaTs) at concentrations shown to be adequate in biofilm eradication. Similarly, here we verified that D-Bac8c^2,5 Leu^ has low toxicity to human monocytes THP-1. Additionally, its hemolytic activity revealed a reduced potential for damaging human red blood cells. Crucially, cytokine production assays demonstrated that D-Bac8c^2,5 Leu^ did not induce the release of pro-inflammatory cytokines in human blood, suggesting a low immunogenic potential. D-Bac8c^2,5 Leu^ inhibited the biofilm-induced increase in IL-8 levels without affecting TNFα, indicating potential anti-inflammatory properties. These combined attributes, as detailed by Zapotoczna et al. (2017), highlight the biocompatibility and therapeutic potential of D-Bac8c^2,5 Leu^, further supporting its safety profile for potential clinical use in wound care.

Though we have mimicked the *in vivo* environment, D-Bac8c^2,5 Leu^ delivery into the wound bed remained a concern. Delivering drugs into the infected wound poses significant challenges due to the presence of biofilms, impaired blood supply, persistent inflammation and high exudate levels, heterogeneous wound tissue composition, and patient-related factors affecting compliance. Inflammatory mediators and proteases in the wound environment can degrade drugs, and wound complexity hampers uniform drug distribution. Overcoming these challenges necessitates innovative non-toxic drug delivery systems capable of optimizing drug efficacy and effective penetration of wound tissue. For that, the incorporation of D-Bac8c^2,5 Leu^ into a MC hydrogel formulation represented a promising approach to overcome these barriers.

MC hydrogels provide a platform for localized and sustained release of the peptide, ensuring continuous delivery directly to the wound site while minimizing systemic exposure. Additionally, hydrogels are reported to enhance the stability of peptides, prolonging their activity and efficacy (Liang et al., 2021; Huan et al., 2022; Casadidio et al., 2023; Jeong et al., 2023). The adherence of hydrogels to the wound bed promotes prolonged contact time, facilitating better interaction between the peptide and bacterial pathogens (Huan et al., 2022; Jeong et al., 2023). Patient comfort and compliance are also improved with hydrogel formulations, as they are typically well-tolerated and easy to apply. The hydrogel formulation used in this study was shown to be stable and thermoresponsive at physiological temperatures (Supplementary Figure S1). Thermoresponsive gels undergo a phase transition in response to temperature changes, typically transforming from a liquid to a gel state upon contact with body temperature (Zhang et al., 2021). This ensures close contact with the wound bed, creating a barrier that seals the wound and prevents the loss of exudate (Marzaman et al., 2022; Wang et al., 2022). This improved contact enhances the delivery of therapeutic agents into the wound bed, optimizing their efficacy in combating infection and promoting wound healing. The efficacy of our loaded gel was tested against planktonic and biofilm growth of the tested strains. The kinetics of bacterial killing exhibited rapid eradication of viable bacteria upon contact with the loaded gel, highlighting its potential as a localized antimicrobial therapy for infected wounds (Figure 5). The release of D-Bac8c^2,5 Leu^ from the MC-*β*-GP gel at concentrations reaching the MIC after 2–3 h of incubation validates the sustained release of the peptide into the surroundings. Notably, the antibiofilm activity of the D-Bac8c^2,5 Leu^-loaded hydrogel against preformed biofilms was less potent compared to free peptide, this is due to the retention of the peptide in the gel where longer exposure time will be needed to establish an enhanced effect. However, a significant reduction in biofilm viable counts demonstrated the effectiveness of the loaded gel in disrupting and eradicating bacterial biofilms. Overall, these results demonstrate the promising potential of D-Bac8c^2,5 Leu^-loaded hydrogel as a novel and effective treatment for chronic wound infections, offering prospect for improved patient outcomes and reduced reliance on traditional antibiotics.

## Data Availability

The original contributions presented in the study are included in the article/Supplementary material, further inquiries can be directed to the corresponding author.
