# Supplementary material for: Potent antimicrobial activity of hydrogel loaded with the antimicrobial peptide, D-Bac8c2,5 Leu, against monospecies and polymicrobial biofilms of Staphylococcus aureus and Pseudomonas aeruginosa
Source: Front Microbiol. 2025 Apr 24;16:1571649. doi: 10.3389/fmicb.2025.1571649 (PMC12058896; doi:10.3389/fmicb.2025.1571649)
Supplement: Supplementary file 1 [file Data_Sheet_1.docx]

**Supplementary Material**

**Table S1. Minimum Inhibitory Concentrations (MIC) of D-Bac8c^2,5 Leu^ against clinical isolates of *S. aureus* and *P. aeruginosa*.**

|  | Clinical Isolate | D-Bac8c^2,5 Leu^ MIC µg/mL |
| --- | --- | --- |
| *Staphylococcus aureus* | MSSA-1 | 8 |
|  | MSSA-2 | 8 |
|  | MSSA-3 | 8 |
|  | MSSA-4 | 8 |
|  | MSSA-5 | 8 |
|  | MSSA-6 | 8 |
|  | MSSA-7 | 8 |
|  | MSSA-8 | 8 |
|  | MSSA-9 | 4 |
|  | MSSA-10 | 4 |
|  | MSSA-11 | 8 |
|  | MSSA-12 | 8 |
|  | MRSA-1 | 16 |
|  | MRSA-2 | 8 |
|  | MRSA-3 | 8 |
|  | MRSA-4 | 8 |
|  | MRSA-5 | 4 |
|  | MRSA-6 | 8 |
|  | MRSA-7 | 8 |
|  | MRSA-8 | 16 |
|  | MRSA-9 | 16 |
|  | MRSA-10 | 8 |
|  | MRSA-11 | 8 |
|  | MRSA-12 | 8 |
| *Pseudomonas aeruginosa* | PA-1 | 16 |
|  | PA-2 | 32 |
|  | PA-3 | 16 |
|  | PA-4 | 16 |
|  | PA-5 | 8 |
|  | PA-6 | 16 |
|  | PA-7 | 16 |
|  | PA-8 | 32 |
|  | PA-9 | 16 |
|  | PA-10 | 16 |

MSSA: methicillin susceptible *Staphylococcus aureus*.

MRSA: methicillin resistant *Staphylococcus aureus*

PA: *Pseudomonas aeruginosa*

**Table S2.** Reduction in Log_10_CFU/mL counts for bacteria treated with D-Bac8c^2,5 Leu^ hydrogel at 512𝜇g/mL versus negative control in static biofilm model.

| **Bacterial Strain** | **Reduction Log_10_CFU/mL ± SD** | **P value**^a^ |
| --- | --- | --- |
| USA300 | 1.515 ± 0.4875 | 0.0359* |
| PAO1 | 0.9874 ± 0.3317 | 0.0409* |
| SH1000 | 4.352 ± 0.3608 | 0.0003*** |
| BH1CC | 1.463 ± 0.2171 | 0.0062** |
| BH48 | 3.321 ± 0.5612 | 0.0041** |

^s^Statistical significance of the difference between means for treated and control groups, determined by unpaired two-tailed t-test. results were shown to be significantly different when comparing treated to untreated biofilm, *** indicating a P < 0.001.

**
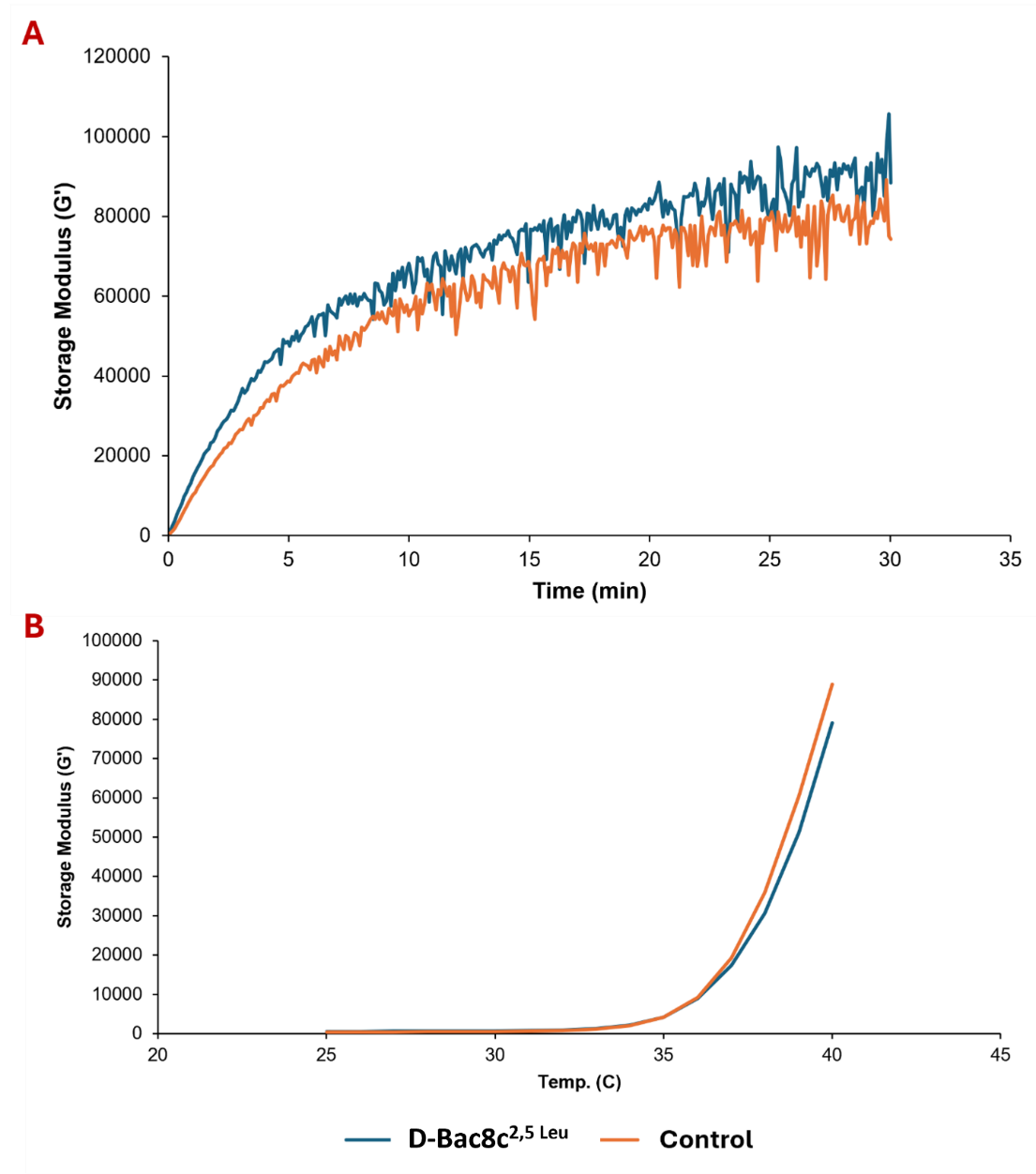
**

**Figure S1. Rheological characteristics of free and loaded 𝛽-GP-Methylcellulose gel.** (A) Oscillatory rheological measurements of the gels taken over a period of 30 minutes at a constant temperature of 37 °C represented as storage modulus G′ versus time. (B) Oscillatory rheological measurements of the gels taken during a temperature sweep from 25 to 40°C represented as storage modulus G′ versus temperature. Data was collected from two independent experiments.


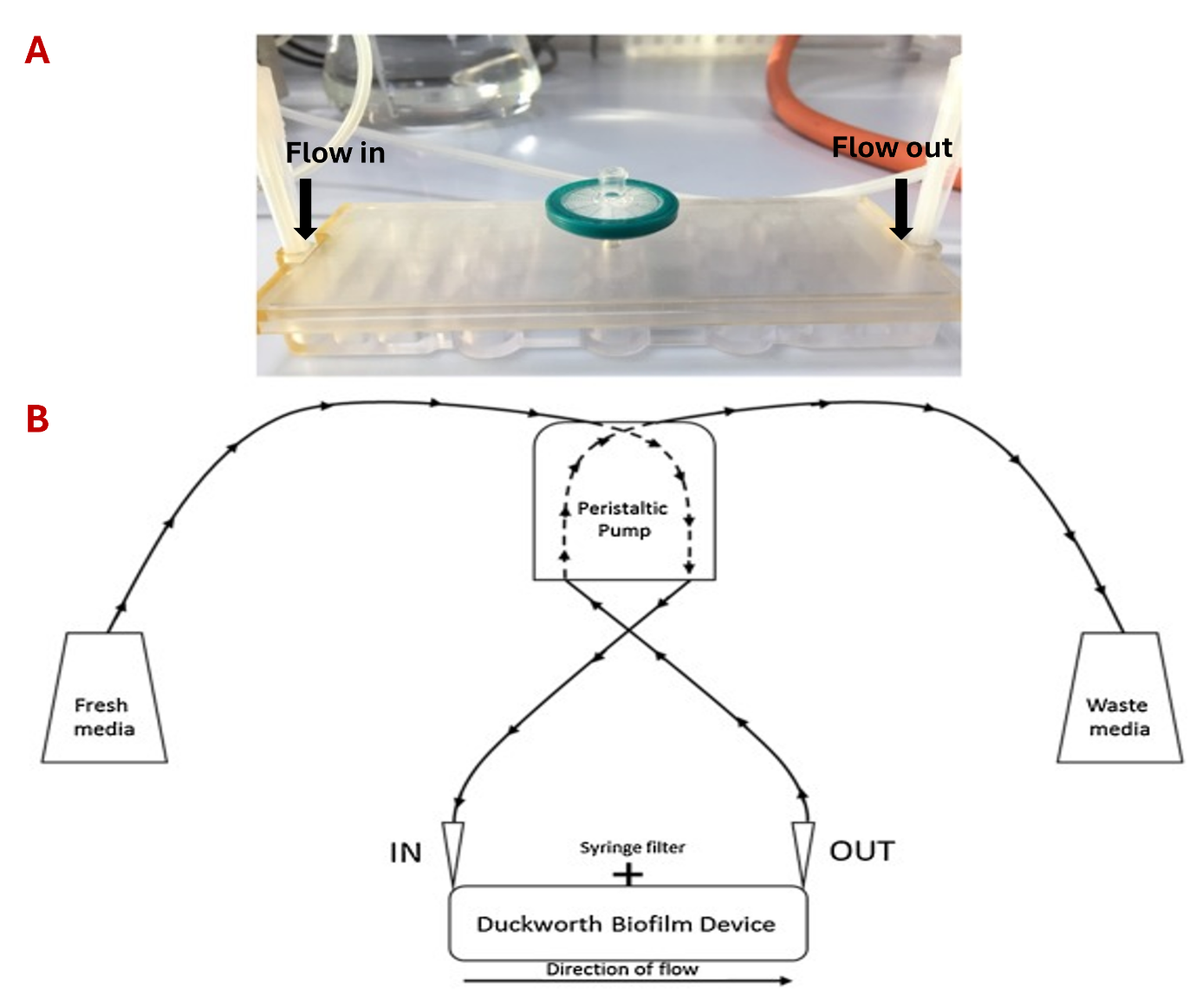


**Figure S2*.* Biofilm formation in duckworth device flow system.** A) Image showing the 3D printed Duckworth deivice and the connected system where the fresh media flow into the device and waste flows out. B)Ilustration of the duckworth system connections.
